# Fluvastatin-induced myofibrillar damage is associated with elevated ROS, and impaired fatty acid oxidation, and is preceded by mitochondrial morphological changes

**DOI:** 10.1038/s41598-024-53446-w

**Published:** 2024-02-09

**Authors:** Mohamed H. Al-Sabri, Nourhane Ammar, Stanislava Korzh, Ahmed M. Alsehli, Kimia Hosseini, Robert Fredriksson, Jessica Mwinyi, Michael J. Williams, Hadi Boukhatmi, Helgi B. Schiöth

**Affiliations:** 1 Department of Surgical Sciences, Division of Functional Pharmacology and Neuroscience, Biomedical Center (BMC), Uppsala University, Husargatan 3, 751 24 Uppsala, Sweden; 2 Department of Pharmaceutical Biosciences, Uppsala University, 751 24 Uppsala, Sweden; 3 Institut de Génétique Et Développement de Rennes (IGDR), Université de Rennes, CNRS, UMR6290, 35065 Rennes, France; 4 Latvian Institute of Organic Synthesis, Aizkraukles 21, Riga, 1006 Latvia; 5 Faculty of Medicine, King Abdulaziz University and Hospital, Al Ehtifalat St., 21589 Jeddah, Saudi Arabia

**Keywords:** Cell biology, Genetics, Molecular biology, Neuroscience

## Abstract

Previously, we showed that fluvastatin treatment induces myofibrillar damage and mitochondrial phenotypes in the skeletal muscles of *Drosophila*. However, the sequential occurrence of mitochondrial phenotypes and myofibril damage remains elusive. To address this, we treated flies with fluvastatin for two and five days and examined their thorax flight muscles using confocal microscopy. In the two-day fluvastatin group, compared to the control, thorax flight muscles exhibited mitochondrial morphological changes, including fragmentation, rounding up and reduced content, while myofibrils remained organized in parallel. In the five-day fluvastatin treatment, not only did mitochondrial morphological changes become more pronounced, but myofibrils became severely disorganized with significantly increased thickness and spacing, along with myofilament abnormalities, suggesting myofibril damage. These findings suggest that fluvastatin-induced mitochondrial changes precede myofibril damage. Moreover, in the five-day fluvastatin group, the mitochondria demonstrated elevated H_2_O_2_ and impaired fatty acid oxidation compared to the control group, indicating potential mitochondrial dysfunction. Surprisingly, knocking down *Hmgcr* (*Drosophila* homolog of *HMGCR*) showed normal mitochondrial respiration in all parameters compared to controls or five-day fluvastatin treatment, which suggests that fluvastatin-induced mitochondrial dysfunction might be independent of Hmgcr inhibition. These results provide insights into the sequential occurrence of mitochondria and myofibril damage in statin-induced myopathy for future studies.

## Introduction

Cardiovascular diseases (CVDs) are the leading causes of mortality globally, accounting for 20.5 million deaths in 2021, with hypercholesteremia as the major risk factor^1,2^. Statins reduce low-density lipoprotein (LDL) cholesterol levels by inhibiting HMG-CoA reductase (HMGCR), the rate-limiting enzyme in the cholesterol biosynthesis pathway in many tissues, including the liver, muscles and brain; therefore, they are the gold standard for the management of CVDs^3–6^. Inhibition of intracellular cholesterogenesis enhances the expression of LDL receptors in hepatocytes, leading to increased clearance of cholesterol from the blood^3^. Despite their beneficial therapeutic effects on the management of CVDs, statins are associated with certain side effects, including muscle damage^7,8^, an increased risk for diabetes^9,10^, cognitive impairment^11,12^, and sleep disturbance^13^. Statin-associated muscle symptoms, otherwise known as statin-induced myopathy (SIM), are the most common side effects of statins, with an incidence of 27.8%, ranging from myalgia to rare muscle necrosis called rhabdomyolysis^14^. SIM compromises patients’ adherence to statin therapy, leading to an elevated risk of developing cardiovascular events^15–17^.

Recent reports have underscored the crucial role of mitochondrial impairment in SIM^7,8,18^. Mitochondrial dysfunction leads to energy depletion and reduced oxidative phosphorylation capacity^7^, diminished mitochondrial membrane potential, activation of apoptosis and ultimately muscle weakness, fatigue and cramps^19–21^. Nevertheless, the role of mitochondrial dysfunction in SIM is still under debate^8,18,22,23^. The exact mechanisms by which statins induce mitochondrial impairment are not fully understood. Studies have indicated potential mechanisms by which statins impact mitochondrial morphology and function^18,24^. For instance, reports have suggested that, due to inhibition of HMGCR, statin treatment leads to a reduced muscular level of coenzyme Q10 (CoQ10), which is a crucial node in the mitochondrial respiratory chain, causing depletion of energy, mitochondrial dysfunction and muscle damage^21,25^. Other studies have shown that statins cause mitochondrial dysfunction through the inhibition of mitochondrial Complexes I, II, and III, which leads to the overproduction of reactive oxygen species (ROS) and cytotoxicity^7^. The inhibition of carnitine-palmitoyltransferase-2 (CPT2) and impairment of fatty acid oxidation (FAO) have also been reported as an underlying cause for statin-induced mitochondrial dysfunction and SIM^7,26^. However, the temporal relationship between mitochondrial dysfunction and muscular damage has not been fully elucidated.

We previously revealed that chronic fluvastatin treatment for five days induced a mitochondrial morphological phenotype (fragmented and round shape) and myofibrillar damage in the skeletal muscles of *Drosophila*^8^. We also demonstrated that targeted knockdown of *Hmgcr* (*Drosophila* homolog of *HMGCR*) in skeletal muscle induced a similar pattern of mitochondrial morphological phenotype (round shape) but was not sufficient to elicit myofibrillar damage^8^. However, the chronic effect of fluvastatin and the role of Hmgcr on mitochondrial respiration function in skeletal muscle are still elusive.

In this study, we used *Drosophila* as a model system to examine whether the fluvastatin-induced mitochondrial phenotype in the skeletal muscles precedes myofibrillar damage and examined the effect of chronic fluvastatin treatment on mitochondrial respiration. To mimic the effect of fluvastatin on its known target, HMGCR, we also investigated whether selective knockdown of *Hmgcr* in skeletal muscles recapitulates fluvastatin's effect on mitochondrial respiration, aiming to expand our understanding of the underlying mechanisms for SIM.

## Results

### Fluvastatin-induced myofibril phenotypes are preceded by mitochondrial morphological changes.

**Figure 1:**
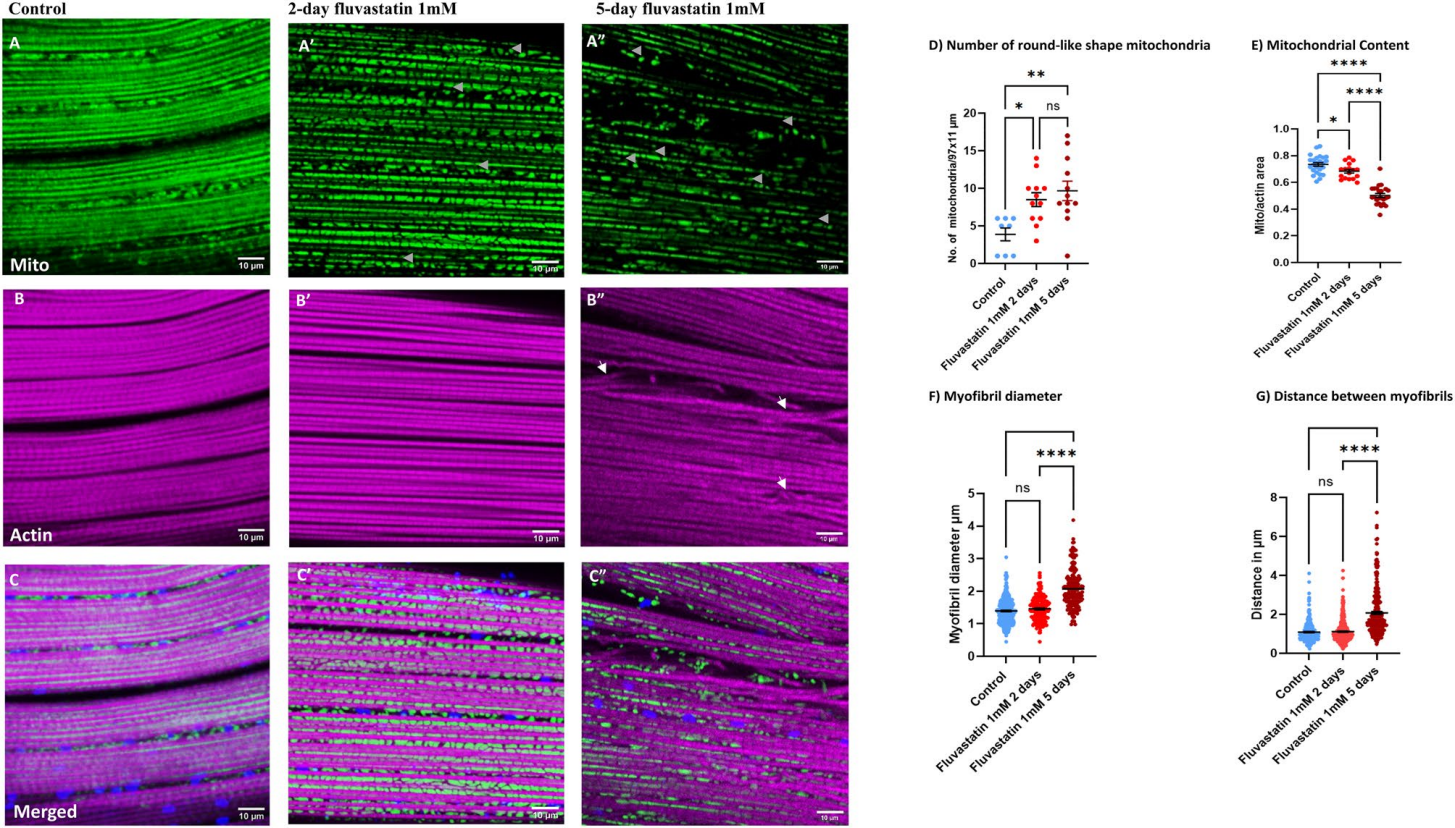
Fluvastatin induces altered mitochondrial morphology that precedes the myofibril abnormalities. *Mef2-GAL4* > *UAS-mitoGFP* thorax flight muscles were stained with phalloidin to visualize actin (magenta), Mef2 labels muscle nuclei (red) and expresses mito-GFP to visualize the mitochondrial matrix (green) under confocal microscopy. In the fluvastatin group, flies were treated with 1 mM fluvastatin in fly food for two or five days while in the control group, flies were fed normal fly food for five days. In the control group, dissected thorax skeletal muscles showed a regular myofibril arrangement along with aggregated and elongated-shaped mitochondria distributed parallel to the myofibril direction (**A**,**B**,**C**). After two days of 1 mM fluvastatin, compared to the control group, skeletal muscles exhibited fragmented mitochondria of different sizes, most of which were round in shape (arrowheads **A’**,**D**) along with organized and parallel myofibrils, which showed normal diameter and spacing (**B’**,**C’**,**F**,**G**). After five days, compared to the control, the skeletal muscles displayed severely aberrant and fragmented mitochondria of small sizes with capacious round shapes (arrowheads **A”**,**D**), and the mitochondria were absent in areas where myofibrils were more disorganized and separated (**A”**,**C”**). The myofibrils also showed abnormal myofilament (arrows **B”**) with longer spacing and thicker diameter compared to the control group (**B”**,**C”**,**B**,**C**,**E**,**F**). The relative mitochondrial content (ratio of mitochondrial area to actin area) was also significantly reduced in the fluvastatin groups in a time-dependent manner. The scale bar is indicated at the bottom of each image. The magnification was 40 megapixels. Each bar represents the mean value (± SEM). *, *P* ≤ 0.05; **, *P* ≤ 0.01**** *P* ≤ 0.0001. The type of the statistical test and number of images used are mentioned in the Method section.

In our previous study, we showed that chronic fluvastatin treatment for five days induced myofibrillar damage and mitochondrial phenotypes (fragmented and round shape) in the skeletal muscles of fly legs^8^. Nevertheless, the sequence of events of mitochondrial alteration and muscle damage remains unknown. To investigate this, we treated adult *Mef2-Gal4* > *UAS-mitoGFP* flies with 1.0 mM fluvastatin for two and five days and used confocal microscopy to examine the morphology of thorax flight muscles. In the control group, the myofibrils were organized in parallel, with normal diameter and spacing and mitochondria (Fig. 1A–C) adopted a filamentous shape, forming an elongated structure along the myofibrils. Interestingly, after two days of fluvastatin treatment, we observed noticeable mitochondrial morphological changes. These changes included a fragmented network of mitochondria with varying sizes and round-like shapes (arrowheads Fig. 1A’,D). Additionally, there was a significant decrease in overall mitochondrial content, as indicated by a reduced ratio of mitochondrial area to actin area (Fig. 1E). However, the myofibrils maintained an organized, parallel pattern, with unaffected diameter and spacing when compared to control flies (Fig. 1B’,C’,F,G).

In chronic five-day fluvastatin treatment, compared to the control, the mitochondria displayed more pronounced fragmentation, with a higher number of round-like shapes, particularly in areas where the myofibrils were more separated (Fig. 1A”,C”,D,E). Additionally, chronic fluvastatin-treated flies showed a more significant reduction in mitochondrial content than both the control and the two-day fluvastatin groups (Fig. 1 E). Intriguingly, the myofibrils exhibited disorganization and separation, characterized by a thicker diameter, significant spacing between individual myofibrils, and abnormal myofilaments, when compared to both the two-day fluvastatin treatment and untreated flies (Fig. 1 arrows, B”, F & G). These observations suggest damage to the myofibrils. Collectively, these findings suggest that fluvastatin treatment induced mitochondrial morphological changes in a time-dependent manner and that these changes preceded myofibril damage in the thorax flight muscles of *Drosophila*.

### Chronic Fluvastatin treatment for five days impairs mitochondrial respiration

**Figure 2:**
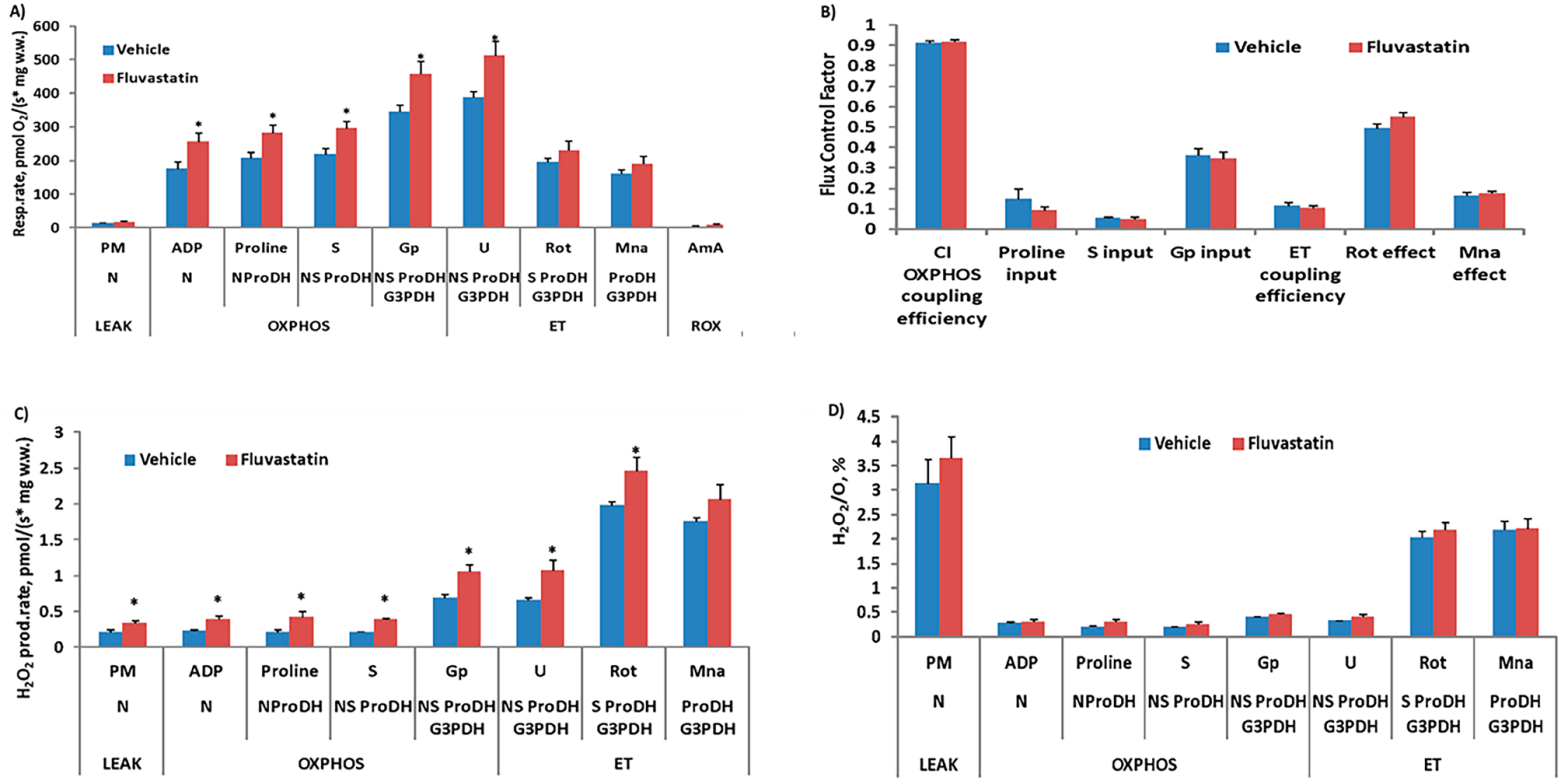
Fluvastatin treatment is associated with increased ROS production. In the fluvastatin group, flies were treated with 1 mM fluvastatin, and then the muscles of the thorax were dissected for the respirometry assay, while in the control group, flies were treated with normal food, both for five days. Each bar represents the mean ± SEM, and the statistical significance was calculated using Student’s t-test whereby *, P ≤ 0.05. N = 5. PM, pyruvate and malate; ADP, adenosine diphosphate; P, pyruvate; S, succinate; Gp, gycerol-3-phosphate; U, uncoupler; Rot, rotenone; AmA, antimycin A; F, fatty acid oxidation-dependent pathway; N, NADH pathway; LEAK, substrate-dependent state; OXPHOS, oxidative phosphorylation-dependent state; ROX, residual oxygen consumption; H2O2, hydrogen peroxide; G3PDH, glyceraldehyde-3-phosphate dehydrogenase; FNS, FAO, NADH and succinate.

Abnormal mitochondrial morphology, as well as content, are associated with impairment of mitochondrial respiration^27,28^. Previous studies have reported that acute fluvastatin treatment for 24 h impairs mitochondrial respiration in myoblast^7^. Since the mitochondria morphological changes including reduced content were dramatically obvious along with severe myofibril damage after chronic fluvastatin treatment (Fig. 1), and studies have linked myofibril damage to excessive production of reactive oxygen species, ROS, and reduced mitochondrial content^29–31^, we sought here to determine whether chronic fluvastatin treatment for five days is also associated with impairment of mitochondrial respiration. To answer this question, we first fed CSORC flies with 1 mM fluvastatin for five days and then dissected thorax skeletal muscles and examined different parameters of mitochondrial respiration using High-Resolution FluoRespirometry^32,33^. Next, we measured mitochondrial respiratory control, specifically the efficiency of the different complexes in the mitochondrial electron transfer system (ETS), using the Substrate-Uncoupler-Inhibitor Titration (SUIT) protocol mentioned in the Methods section of this study. Fluvastatin treatment significantly increased the respiration rate at all measured pathway-linked OXPHOS states and at the NSProDHGp-linked ET state (Fig. 2A), while calculated flux control factors remained unchanged, indicating that treatment with fluvastatin did not affect the activity/capacity of electron transfer system complexes (Fig. 2B). In the fluvastatin-treated group, an increase in the H_2_O_2_ production rate was observed (Fig. 2C), while the H_2_O_2_/O ratio remained comparable to that of the control group (Fig. 2D), suggesting that the observed increase in the respiration rate in the statin-treated group is more likely a result of an increased H_2_O_2_ production rate. Overall, these results indicate that fluvastatin treatment increases ROS production without affecting individual complexes of the electron transfer system.

**Figure 3:**
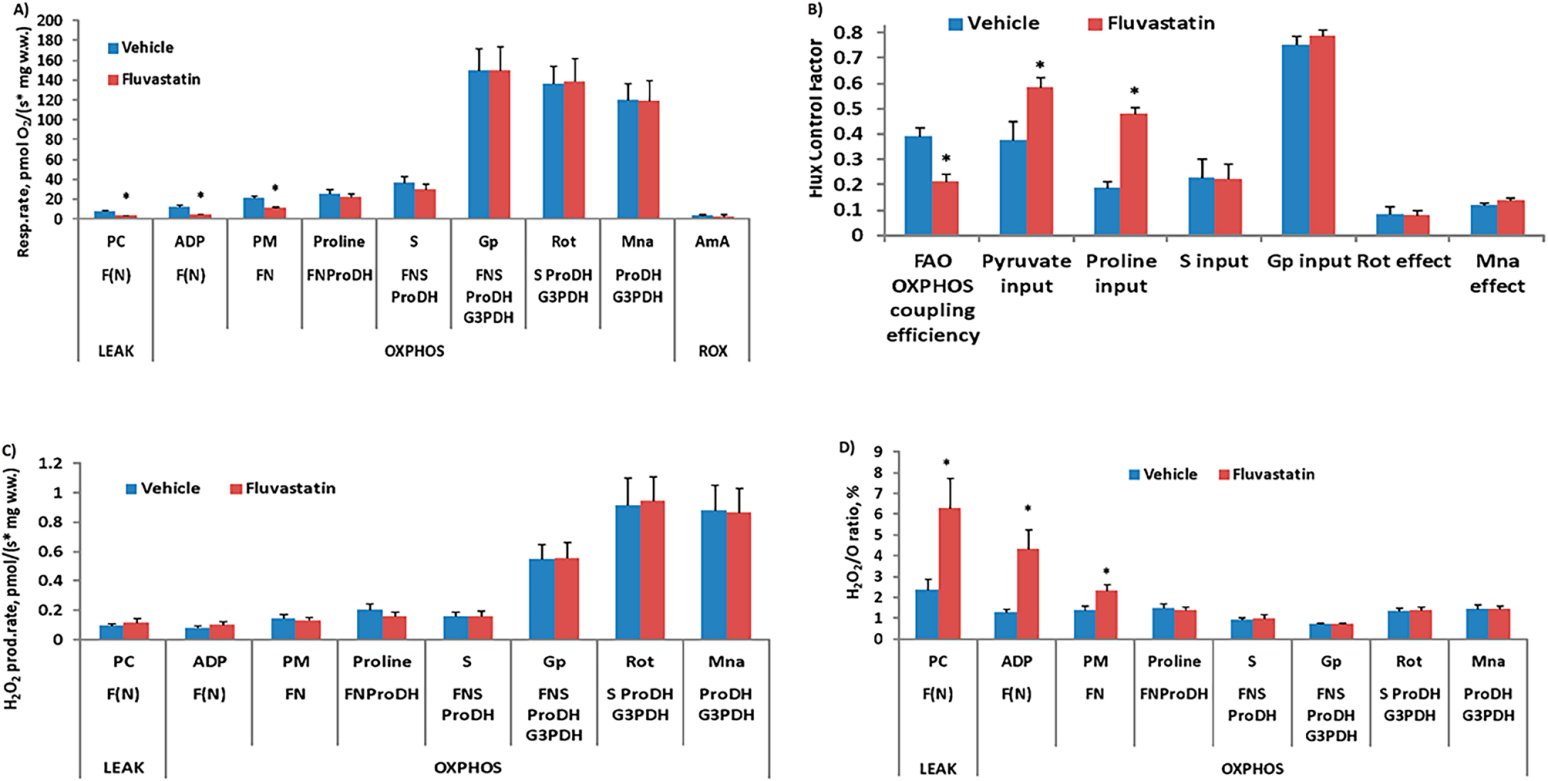
Fluvastatin treatment is associated with decreased CPT2-dependent fatty acid oxidation. In the fluvastatin group, flies were treated with 1 mM fluvastatin-supplemented food, and then the muscles of the thorax were dissected for the respirometry assay, while in the control group, flies were treated with normal food, both for five days. Each bar represents the mean ± SEM, and the statistical significance was calculated using Student’s t-test whereby *, P ≤ 0.05. N = 5. PC, palmitoylcarnitine; ADP, adenosine diphosphate; PM, pyruvate and malate; P, pyruvate; S, succinate; Gp, Glycerol-3-phosphate; U, uncoupler; Rot, rotenone; AmA, antimycin A; F, fatty acid oxidation-dependent pathway; N, NADH pathway; LEAK, substrate-dependent state; OXPHOS, oxidative phosphorylation-dependent state; ROX, residual oxygen consumption; H2O2, hydrogen peroxide; G3PDH, glyceraldehyde-3-phosphate dehydrogenase; FNS, FAO, NADH and Succinate.

We previously showed that chronic fluvastatin treatment is associated with lipotoxicity through disruption of the key genes involved in lipid metabolism^8^. Thus, we sought to determine whether chronic fluvastatin treatment for five days impairs the mitochondrial fatty acid of the thorax muscles. Interestingly, in the fluvastatin-treated group, FAO-dependent respiration at LEAK and OXPHOS states (Fig. 3A), as well as FAO-dependent OXPHOS coupling efficiency (Fig. 3B), were significantly decreased when compared with the control. A lower respiration rate was also observed at the FN-pathway-linked OXPHOS state, while afterwards, the respiration rate of the fluvastatin-treated group was comparable with control values (Fig. 3A). Flux control factors were higher for pyruvate and proline input in the fluvastatin group compared with the control group (Fig. 3B), indicating that fluvastatin-induced stimulation of pyruvate and proline-related metabolism compensates for the reduction in FAO (CPT2-dependent) for ATP production. In addition, an increase in the H_2_O_2_/O ratio was observed after treatment with fluvastatin when compared with the control (Fig. 3C), suggesting that part of the consumed oxygen is wasted for ROS production. Overall, these results suggest that treatment with fluvastatin induces a decrease in CPT2-dependent FAO, with a concomitant increase in pyruvate and proline pathways, while electron transfer system efficiency was altered. In agreement with these results, fluvastatin has been shown to reduce the activity of Complexes I & II without affecting the activity of Complex III^7^. Other reports have also revealed that the inhibition of CPT2 and impairment of FAO are also associated with SIM^7,26^.

### *Hmgcr* knockdown in skeletal muscles does not alter mitochondrial respiration

The statin's main target is HMGCR. Thus, we asked whether fluvastatin-induced mitochondrial dysfunction resulted from the inhibition of HMGCR. To mimic the statin inhibitory effect on HMGCR enzyme activity, we performed a specific knockdown of *Drosophila Hmgcr* in the skeletal muscles using the *Mhc-Gal4* driver. Then, we dissected thorax skeletal muscles and examined different parameters of respiration using High-Resolution FluoRespirometry. Surprisingly, compared with both controls (*Mhc-Gal4* > *w*^*1118*^* & w*^*1118*^ > *UAS-GAL4 RNAi*), *Hmgcr* knockdown flies (*Mhc-Gal4* > *UAS Hmgcr RNAi*) showed a normal respiration rate at all measured pathway-linked OXPHOS states and at the NSProDHGp-linked ET state (Supplementary Fig. 1A), as well as flux control factors remained unchanged (Supplementary Fig. 1B). This indicates that knockdown of *Hmgcr* in skeletal muscles may not affect the mitochondrial activity/capacity of any electron transfer system complexes (Supplementary Fig. 1B). In addition, no changes in the H_2_O_2_ production rate (Supplementary Fig. 1C) or the H_2_O_2_/O ratio (Supplementary Fig. 1D) were observed compared with controls. Overall, these results suggest that the inhibition of Hmgcr in skeletal muscles does not affect the respiration rate, ROS production or individual complexes of the electron transfer system of *Drosophila* mitochondria.

In terms of fatty acid oxidation, we noticed that in *Hmgcr* knockdown flies, FAO-dependent respiration at LEAK and OXPHOS states (Supplementary Fig. 2A), as well as FAO-dependent OXPHOS coupling efficiency (Supplementary Fig. 2B), were not changed compared with both controls. A normal respiration rate was also observed at FN-pathway linked OXPHOS state, and the respiration rate in the fluvastatin-treated group was comparable to the control values (Supplementary Fig. 2A). Flux control factors were also normal for pyruvate and proline input in the *Hmgcr* knockdown flies compared with controls (Supplementary Fig. 2B). In addition, no change in the H_2_O_2_/O ratio was observed in the *Hmgcr* knockdown flies (Supplementary Fig. 2C). Altogether, these results suggest that *Hmgcr* knockdown in skeletal muscles did not alter CPT2-dependent FAO or result in changes in electron transfer system efficiency.

## Discussion

In *Drosophila* skeletal muscles, compared with the control, acute fluvastatin treatment for two days, mitochondria were fragmented with a higher number of round-like shapes and their content was significantly reduced, all without noticeable alterations in myofibrils. With chronic fluvastatin treatment for five days, the mitochondria exhibited extensive fragmentation, notable rounding up and a more significant reduction in the mitochondria content. However, these mitochondrial phenotypic changes were accompanied by a noticeable disorganization of myofibrils, characterized by a thicker diameter and greater spacing between them along with myofilament abnormalities. These findings may suggest that fluvastatin-induced myofibril damage is proceeded by mitochondrial morphological phenotypes.

A recent study has indicated coordination between mitochondrial shape and myofibril tension in *Drosophila* flight thorax muscles^34^. The same study has shown that in normal muscles, the area where the myofibrils are intact, the mitochondria adopt an elongated shape oriented in the direction of the myofibrils^34^. On the other hand, in the area where the myofibrils are mechanically severed, mitochondria exhibit rounded shapes, especially when the connection between the mitochondria and myofibril is lost^34^. In this context, we demonstrated here that mitochondria displayed fragmentation and rounding up which became more pronounced over time with fluvastatin treatment, especially in the area where the connection between myofibrils and mitochondria was lost, suggesting a temporal relation between mitochondrial morphological alteration and myofibril damage. Given this, two possibilities are proposed: There is a bidirectional feedback mechanical cue, or a default of one cell component (mitochondria) may cause a morphological defect to the other (myofibril). This can be a subject of future investigations.

Mitochondrial content refers to the amount of mitochondrial proteins and DNA and reflects the number of mitochondria in the muscle fibres^34,35^. This is crucial to allow the muscles to generate more ATP to meet energy demand, as mitochondrial respiration is contingent upon mitochondrial content^27,28^. Acute exposure to fluvastatin for 24 h has been reported to impair mitochondrial respiration^7^ and induce apoptosis^36^ in different cell types. Muscle fibre damage can lead to excessive ROS production, which, in turn, triggers further degradation of mitochondrial content, including mitochondrial proteins, enzymes and genetic materials^29–31^. Consistent with this, we reported here that chronic fluvastatin treatment for five days caused excessive ROS production (see Fig. 2), which may consequently exacerbate mitochondrial degradation and content reduction (see Fig. 1 E). This may explain the reduced locomotion and climbing activity associated with chronic fluvastatin treatment in flies reported previously^8^. In humans, several statins have been shown to alter mitochondrial respiration acutely and chronically^7^, inducing ROS overproduction and subsequent oxidative stress in skeletal muscle^18,19,37^, and reducing mitochondrial content^24,38,39^.

Mitochondria and myofibrils are connected through the sarcoplasmic reticulum (SR), which serves as a calcium store^40,41^. Intriguingly, statins have been demonstrated to alter SR calcium ATPases and cause disconnection of the stabilizing protein FK506 binding protein (FKBP12) from the calcium release channel, Ryanodine Receptor (RyR), in the SR, which is accompanied by ROS^42,43^. Interestingly, these proteins are regulated by mitochondrial ROS^44^. Here, we noted that chronic fluvastatin treatment was accompanied by elevated ROS levels. Although chronic fluvastatin has been reported to impair the SR/mitochondria calcium store in rat skeletal muscle^45^, it would be interesting to examine the acute effect of fluvastatin on SR proteins at different time points and thus understand whether SR damage causes or is initiated by mitochondrial dysfunction and leads to subsequent separation of myofibrils and mitochondria.

We previously demonstrated that *Hmgcr* knockdown in skeletal muscles in *Drosophila* induces rounding up of mitochondria similar to chronic five-day fluvastatin treatment, but it was not sufficient to trigger myofibril damage^8^. Accordingly, we hypothesized that fluvastatin treatment induced mitochondrial dysfunction through the inhibition of Hmgcr in skeletal muscles, leading to subsequent ROS overproduction. To investigate this hypothesis, we measured and compared mitochondrial respiration for chronic fluvastatin treatment and selective *Hmgcr* knockdown in thorax flight muscles. Contrary to our initial hypothesis, *Hmgcr* knockdown did not alter the respiration rate at any of the measured pathways, nor did it lead to changes in ROS production or CPT2-dependent FAO compared to controls or chronic fluvastatin treatment (See Fig. 2 & 3 and Supplementary Fig. 1 & 2). This may indicate that fluvastatin-induced mitochondrial dysfunction is likely independent of Hmgcr inhibition. It is plausible to suggest that the depletion of CoQ10, which is one of the downstream products of the mevalonate pathway, may not be the primary underlying cause of statin-induced mitochondrial dysfunction and subsequent ROS overproduction. In support of this possibility, several publications have indicated that statins cause myopathy through inhibition of mitochondrial repatriation without altering the muscular CoQ10^46,47^. This may explain why CoQ10 does not improve the mitochondrial alteration in cell models treated with statins^48^, and CoQ10 supplementation in statin-treated patients has been shown to not improve myopathy-related symptoms^46,49^. However, this does not necessarily exclude the possibility that Hmgcr inhibition in the muscles contributes to the rounded shape phenotype of mitochondria, as we previously showed^8^. Instead, it suggests that the round-up phenotype of mitochondria may be mediated independently of mitochondrial respiration.

We also previously reported that in *Drosophila*, fluvastatin-induced muscular damage is mediated by the inhibition of the muscular chloride channel and is independent of muscular Hmgcr inhibition in skeletal muscles^8^. In the same study, we indicated that chronic fluvastatin treatment was associated with the disruption of key genes involved in lipid metabolism^8^. In our mechanistic model, we suggested that fluvastatin activation of Pkcdelta (*Drosophila* homolog of PKCθ, which upon activation inhibits the chloride channel of skeletal muscles in humans and rats^50,51^) is mediated through both ROS and free fatty acids, FFAs^8^. Here, we demonstrated that chronic fluvastatin treatment led to elevated ROS in skeletal muscles and impaired CPT2-dependent FAO in skeletal muscles. CPT2 is a crucial enzyme for mitochondrial uptake of FFAs from the cytosol into mitochondria and converts fatty acylcarnitines to acyl-CoA for β-oxidation^7,52^. Impairment of CPT2 activity has been reported to accumulate intramuscular and serum-free fatty acids and acylcarnitines^53–55^. Several statins have been associated with the inhibition of CPT2^18,56^ and increased levels of both FFAs and acylcarnitines^10,57^. FFA and acylcarnitine are strong activators of PKCθ in the muscles^58,59^. Based on this, fluvastatin-induced impairment of CPT2-dependent FAO in the skeletal muscles may lead to elevated FFAs and acylcarnitines, which, in addition to ROS, contribute to activated Pkcdelta-mediated inhibition of skeletal muscle chloride channels and subsequent myopathy-like phenotypes.

The question that remains is how fluvastatin elicits mitochondrial dysfunction. Two potential mechanisms have been proposed in the literature. First, direct inhibition of mitochondrial complexes has been reported with several statins, including fluvastatin, in humans^7,60^ although this has not been investigated in flies yet. A study by Schirris et al. has indicated that most of the statin-lactone forms reduce the mitochondrial respiration capacity and are associated with inhibition of mitochondrial Complex I, II, and III. The same study showed that the activity of mitochondrial Complex III was reduced in C2C12 myoblast where the Q_o_ binding site of mitochondrial Complex III was identified as off-target of the statin lactones^7^. However, fluvastatin demonstrated a strong inhibitory effect on Complex I and II with no effect on Complex III, which was attributed to the intermediate lipophilicity of fluvastatin, although other hydrophilic statins showed to inhibit Complex III^7^. Taken together with our findings, fluvastatin might impair mitochondrial respiration through inhibition of Complex I and II.

Another possibility is that fluvastatin-induced mitochondrial dysfunction is mediated by lipotoxicity. Here, we demonstrated that fluvastatin treatment was associated with impairment of CPT2 which may result in reduced uptake and accumulation of FFA. In addition, in our previous study, we demonstrated that fluvastatin treatment is associated with disruption of key genes involved in lipid metabolism, which may also exacerbate the accumulation of toxic lipid intermediates such as FFA and diacylglycerides (DAGs) in the muscles. Of interest, we previously found that Lipn (*Drosophila* homolog of *LPIN1* which encodes lipin-1 enzymes, responsible for the dephosphorylation of phosphatidic acid into DAGs*)* was highly significantly downregulated in fluvastatin-treated flies^8^*.* In support of this, statins have been shown to reduce the expression of LIPN1 in the skeletal muscles of mice. The same study indicated that loss of LIPN1 in the skeletal muscles leads to aberrant lipid storage, impaired mitochondrial function and ultimately myopathy^61^.

This is the first study using *Mef2-Gal4* > *UAS-mitoGFP*, which helped visualize and analyse phenotypic changes in the mitochondria in a time-dependent manner under confocal microscopy. This line can be used further to investigate mitochondrial phenotypes associated with other types of statins. Additionally, we quantified the mitochondrial and myofibril phenotypes, which strengthened our findings. We measured and compared respiration functions for both fluvastatin-treated and selective skeletal muscular *Hmgcr* knockdown flies, allowing us to elucidate the known statin-target effect of Hmgcr on muscular mitochondria. Nevertheless, there are certain limitations in our study. Although Acute exposure for 24 h to fluvastatin has been shown to impair mitochondrial respiration^7^ and induce apoptosis^36^ in different cell types, it would be interesting to investigate the effect of fluvastatin on mitochondrial respiration functions after two days in *Drosophila*. Although all statins share a common target, HMGCR and studies showed that both, lipophilic and hydrophilic statins can penetrate mitochondria and impair mitochondrial respiration^7,18^, we could not generalize the effect of the fluvastatin (which is moderately lipophilic) on temporal dynamics of mitochondrial and myofibril damage on other hydrophilic statins. Despite the fact that fluvastatin induces apoptosis in different study models^7,36,62,63^, measuring apoptotic markers, such as caspase activation, and cytochrome C release, in the skeletal muscles of *Drosophila* would enhance our understanding of whether the mitochondrial phenotypes associated with fluvastatin represent the initiation of cell death cascades, subsequently resulting in myofibril damage. Also, we did not examine the effect of fluvastatin at different time points on the sarcoplasmic reticulum (SR) and its proteins involved in calcium regulation, such as SERCA (Sarco(endo)plasmic Reticulum Calcium ATPase) and RyR. Finally, we did not rescue the mitochondrial phenotypes associated with fluvastatin treatment or assess whether CoQ10 or mevalonate can rescue statin-associated mitochondrial dysfunction, although specific Hmgcr inhibition in skeletal muscles provides us with relevant information regarding the depletion of mevalonate and CoQ10.

## Conclusion

To our knowledge, this is the first study providing insights into the sequential occurrence of fluvastatin-induced mitochondrial morphological changes and myofibril damage in *Drosophila*. We demonstrate that fluvastatin reduces mitochondrial content in a time-dependent manner and that fluvastatin-induced mitochondrial phenotypes precede myofibril damage. Furthermore, we show that chronic fluvastatin treatment impairs mitochondrial respiration, particularly by inducing elevated ROS and impaired FAO, while skeletal muscle Hmgcr knockdown does not lead to altered mitochondrial respiration. Further investigations are warranted to explore the effect of the statins on the sarcoplasmic reticulum at different time points which may link early mitochondrial alteration to later myofibril damage.

## Materials and methods

### *Drosophila* work

To characterize the effect of fluvastatin on mitochondrial morphology, we first decorated actin and mitochondria in skeletal muscles by expressing GFP fused to a mitochondrial matrix targeting signal (*UAS-mito-GFP*, Bloomington #8442) with the *Mef2-GAL4* driver (Bloomington #25756) to generate *Mef2-Gal4* > *UAS-mitoGFP* fly lines. Previous publications have shown that fluvastatin treatment is effective in flies and able to inhibit Hmgcr^8,64–67^. In our previous study, we showed that 1.0Mm fluvastatin, which is within the therapeutic range of fluvastatin used in humans and able to induce mitochondrial phenotypic changes^8^. Therefore, we used 1.0 mM fluvastatin (Sigma, Darmstadt, Germany, #SML0038) in this study as well. Accordingly, we treated *Mef2-Gal4* > *UAS-mitoGFP* flies with 1.0 mM fluvastatin mixed with standard fly food medium (treated group), while in the control, flies were fed with normal standard fly food medium mixed with an equivalent volume of water for two and five days.

### Immunohistochemistry and microscopy

For the imaging experiments, we did not observe any sex-based difference in measurements, therefore we used both male and female *Mef2-Gal4* > *UAS-mitoGFP* flies, which is aligned with a previous study that used the same line^34^*. Mef2-Gal4* > *UAS-mitoGFP* flies (both male and females) were anaesthetized by CO2, and their adult thorax muscles, which are commonly used in examining mitochondrial morphology and respiration^32,34^, were dissected and stained as described in Hunt and Demontis^68^. We used rabbit anti-Mef2 (1:200, Eileen Furlong, Heidelberg, Allemagne), Alexa-conjugated phalloidin (1:200, Thermo Fisher, Waltham/Massachusetts) and goat anti-GFP (1:200, Abcam, ab6673). Samples were acquired on Leica SP5 microscopes (Biosit, IGDR-Rennes) at 40X magnification and 1024/1024-pixel resolution. Images were processed with ImageJ software.

### Quantification of mitochondrial content

The quantification of mitochondrial content was carried out according to Avellaneda et al.^34^*.* The total areas of flight muscle (phalloidin) and mitochondria (mito-GFP) were quantified via Otsu thresholding in ImageJ Fiji for each acquisition channel. This was performed for the same Z-stack number for each condition. The significant difference between groups was calculated using one-way ANOVA with Bonferroni’s multiple comparisons. The number of images used is 15 for the control, 17 for the two-day fluvastatin group and 23 for the five-day fluvastatin group.

### Quantification of the number of round-like shaped mitochondria

Three confocal images of the dissected thorax flight muscles were selected randomly per group. Next, using ImageJ Fiji, the mitochondria channel (mito-GFP) was selected, followed by segmenting the image into six slices using the "Plugins" and "Macros" menu, "StartUp Macros" option. We applied the following script:

N = 6;

w = getWidth;

h = getHeight;

id = getImageID;

for(i = 0; i < N; i + +){

   selectImage(id);

   makeRectangle(0, i*h/N, w, h/N);

   name = "" + (i + 1);

   run("Duplicate…", "title = " + name);

#### }

Afterwards, we counted the round-like mitochondria in each slice using the multipoint tool. Slices where no round-like shapes were found were excluded from the analysis. Subsequently, we used GraphPad Prism to check the normality of the data and select an appropriate test to calculate the significance.

### Measuring the distance between myofibrils

Six random confocal images of dissected thorax flight muscles were selected for each group. Using ImageJ Fiji, actin (phalloidin) and mitochondria channels (mito-GFP) were selected, and then the distance between myofibrils was measured manually. In cases where myofibrils were not consistently aligned or parallel, we measured the distance between myofibrils three times: at the beginning, in the middle, and at the end of the individual pair of myofibrils. Areas where mitochondria were absent or where nuclei between individual pairs of myofibrils were present were excluded from the analysis. We used GraphPad Prism to check the normality of the data and select an appropriate test to calculate the significance.

### Measuring the thickness of myofibrils

Five random confocal images of dissected thorax flight muscles were chosen randomly for each group. Then, we measured the thickness of individual myofibrils using ImageJ Fiji. In cases where the myofibril thickness exhibited variation across the image, we conducted measurements in both the thicker and thinner sections of each myofibril. Subsequently, we used GraphPad Prism to check the normality of the data and select an appropriate test to calculate the significance.

### High-resolution FluoRespirometry

To assess the influence of fluvastatin on mitochondrial respiration, we used wild-type male CSORC flies that were generated by crossing CantonS and OregonR-C flies, both of which were ordered from Bloomington Stock Centre (Bloomington, IN, USA). We first fed CSORC flies with 1.0 mM fluvastatin for five days as described in our previous study^8^ and then dissected the thorax muscles. The knockdown of *Hmgcr* was carried out by the UAS-Gal4 System as described in our previous study^8^ in which *Mhc-Gal4* > *UAS-Hmgcr RANi* is the *Hmgcr* knockdown flies, while *w1118* > *UAS-Hmgcr RNAi* & *Mhc-Gal4* > *w1118* are the control groups^8^. The *Hmgcr* knockdown efficiency of the line was also determined in our previous study^8^. Only male flies were collected and used in this study and the life span of most *Hmgcr* knockdown flies was short, less than 2 days, which is consistent with our previous study^8^. The lines used for knockdown were obtained from Bloomington Stock Center as follows: Mhc-Gal4 (BN 38,464):w[*]; P{w[+ mC] = Mhc-RFP.F3-580}2, P{w[+ mC] = Mhc-GAL4.F3-580}2/SM6b and UAS-Hmgcr RNAi (BN 50,652): y[1] v[1]; P{y[+ t7.7]v[+ t1.8] = TRiP.HMC03053}attP40.

As described in a previous study, permeabilized thoraxes were prepared from four to five flies with slight modifications^69^. Briefly, dissected thoraxes were permeabilized with 62.5 µg/mL saponin at 4 °C for 15 min in preservation buffer (20 mM imidazole, 0.5 mM dithiothreitol, 20 mM taurine, 7.1 mM MgCl_2_, 50 mM MES, 5 mM ATP, 15 mM phosphocreatine, 2.6 mM CaK_2_EGTA, 7.4 mM K_2_EGTA, pH 7.0 at 0 °C). Then, permeabilized thoraxes were washed once for 10 min in MiR05 medium (110 mM sucrose, 60 mM K-lactobionate, 0.5 mM K_2_EGTA, 3 mM MgCl_2_, 20 mM taurine, 10 mM KH_2_PO_4_, 20 mM HEPES, pH 7.1, 0.1% BSA (FA-free), pH 7.1 at 30 °C). Approximately 1 mg of tissue was used for one experimental run. Mitochondrial respiration and H_2_O_2_ production measurements were performed at 25 °C using Oxygraph-2k (O2k; Oroboros Instruments, Innsbruck, Austria) with O2k-Fluo-Modules in MiR05 medium, oxygenated to 400–450 µM to avoid any potential oxygen limitation to respiration. H_2_O_2_ flux was measured simultaneously with respirometry in the O2k-Fluorometer using the H_2_O_2_-sensitive probe Ampliflu™ Red (AmR) as previously described^58^. The H_2_O_2_/O flux ratio [%] was calculated as the H_2_O_2_ flux/(0.5 O_2_ flux).

### Substrate-Uncoupler-Inhibitor Titration

Pyruvate and malate (10 mM and 2 mM, respectively) were used to determine the NADH (N)-pathway and Complex I (CI)-linked LEAK respiration. Adenosine diphosphate (ADP) was added at a 5 mM concentration to determine oxidative phosphorylation-dependent respiration (OXPHOS state). Then, proline (5 mM, proline dehydrogenase substrate, ProDH-pathway) was added to measure NProDH-pathway-linked respiration. Next, succinate (10 mM, Complex II (CII) substrate, S-pathway) was added to reconstitute convergent NSProDh-pathway, CI-&CII- (CIII-linked) and ProDH-linked respiration. Glycerol-3-phosphate (15 mM, glycerol-3-phosphate dehydrogenase substrate, Gp-pathway) was then added to measure NSProDHGp pathway-linked respiration in the OXPHOS state. Titration with the uncoupler carbonyl cyanide m-chlorophenyl hydrazine (CCCP) (0.5 µM steps) was performed to determine the electron transfer system (ETS) capacity. Next, rotenone (0.5 µM, inhibitor of Complex I) and malonate (5 mM, inhibitor of Complex II) were subsequently added to determine SProDHGp- and ProDHGp-linked ETS capacity. Finally, antimycin A (2.5 µM, inhibitor of Complex III) was added to measure nonmitochondrial residual oxygen consumption (ROX).

### Fatty acid oxidation

FAO-dependent mitochondrial respiration in fibres was measured using palmitoylcarnitine (10 µM) and 0.1 mM malate as substrates. ADP was added to a concentration of 5 mM to assess respiration in the OXPHOS state. Pyruvate (5 mM, CI substrate, NADH (N)-pathway) and additional malate (to a final concentration of 2 mM) were then added to reconstitute FN pathway-linked respiration. Then, proline (5mM), succinate (10 mM) and glycerol-3-phosphate (15 mM) were subsequently added to the chamber to measure FNProDH-, FNSProDH- and FNSProDHGp-pathway-linked respiration rates. Next, the inhibitors rotenone and malonate were added to determine SProDHGp- and ProDHGp-linked respiration rates in the OXPHOS state. Antimycin A was added to determine non-mitochondrial residual oxygen consumption (ROX).

To determine the contribution of each substrate to the respiration rate as well as the OXPHOS and ET coupling efficiencies, the flux control factor was calculated as follows:

$$1-\frac{Resp.rate\,before\,the\,addition}{Resp.rate\,after\,the\,addition}.$$

### Statistical analysis

Data obtained from each experiment was first checked for normality using the Shapiro–Wilk test. If the data is normally distributed, a parametric test was used. If the data is not normally distributed, a non-parametric test is used. Accordingly, for non-normally distributed data the statistical significance was calculated with a nonparametric analysis using the Kruskal‒Wallis test followed by the Dunn test for multiple comparisons in Figs. 1D,F,G. For normally distributed data, one-way ANOVA with Bonferroni's multiple comparisons test was used in Fig. 1E and supplementary Figs. 1 and 2. A Student t-test was used in Figs. 2 and 3 to calculate the significance difference.

### Supplementary Information

Supplementary Information.

## Data Availability

All data are contained within the article and supplementary data.
