# Supplementary material for: Fluvastatin-induced myofibrillar damage is associated with elevated ROS, and impaired fatty acid oxidation, and is preceded by mitochondrial morphological changes
Source: Sci Rep. 2024 Feb 9;14:3338. doi: 10.1038/s41598-024-53446-w (PMC10858229; doi:10.1038/s41598-024-53446-w)
Supplement: Supplementary file 1 — Supplementary Information. [file 41598_2024_53446_MOESM1_ESM.pdf]

Fluvastatin-Induced Myofibrillar Damage Is Associated with Elevated ROS, and Impaired Fatty Acid Oxidation, and is Preceded by Mitochondrial Morphological Changes

Mohamed H. Al-Sabri[1,2*], Nourhane Ammar[3†], Stanislava Korzh[4†], Ahmed M. Alsehli[1,5], Kimia Hossiene[2], Robert Fredriksson[2], Jessica Mwinyi[1], Michael J. Williams[1], Hadi Boukhatmi[3], Helgi B. Schiöth[1*]

Supplementary Figures

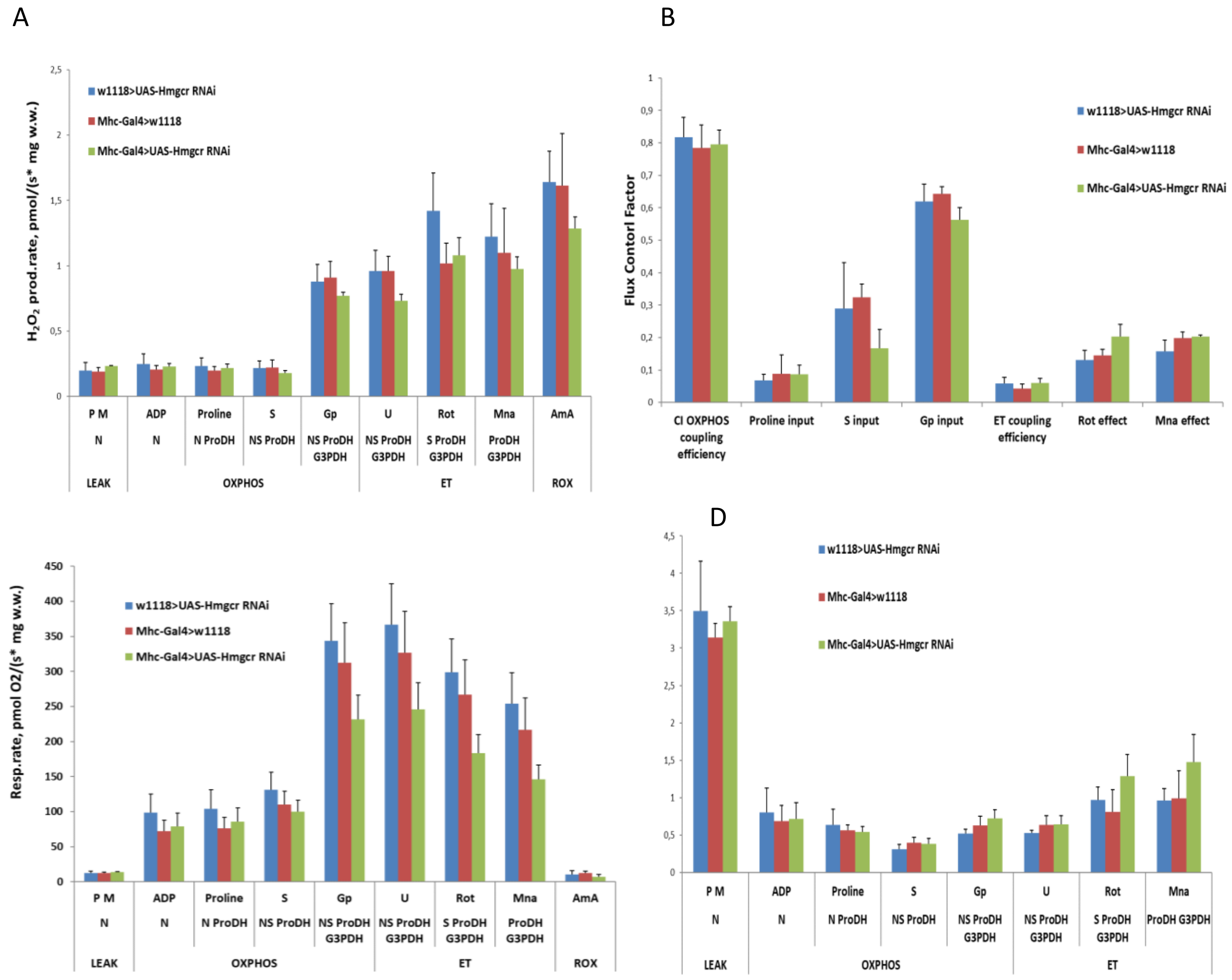

Supplementary Fig. 1: *Hmgcr* knockdown in skeletal muscles does not induce ROS overproduction or impair respiration. The thorax muscles were dissected for the respirometry assay, where *Mhc-Gal4>UAS-Hmgcr RANi* is the *Hmgcr* knockdown flies, while *w1118>UAS-Hmgcr RNAi* & *Mhc-Gal4>w1118* are the control groups. Each bar represents the mean ± SEM, and the statistical significance was calculated using one-way ANOVA with Bonferroni's multiple comparisons test. N=5. PM, pyruvate and malate; ADP, adenosine diphosphate; P, pyruvate; S, succinate; Gp, glycerol-3-phosphate; U, uncoupler; Rot, rotenone; AmA, antimycin A; H2O2, hydrogen

peroxide; G3PDH, glyceraldehyde-3-phosphate dehydrogenase; F, fatty acid oxidation-dependent pathway; N, NADH pathway; LEAK, substrate-dependent state; OXPHOS, oxidative phosphorylation-dependent state; ROX, residual oxygen consumption.

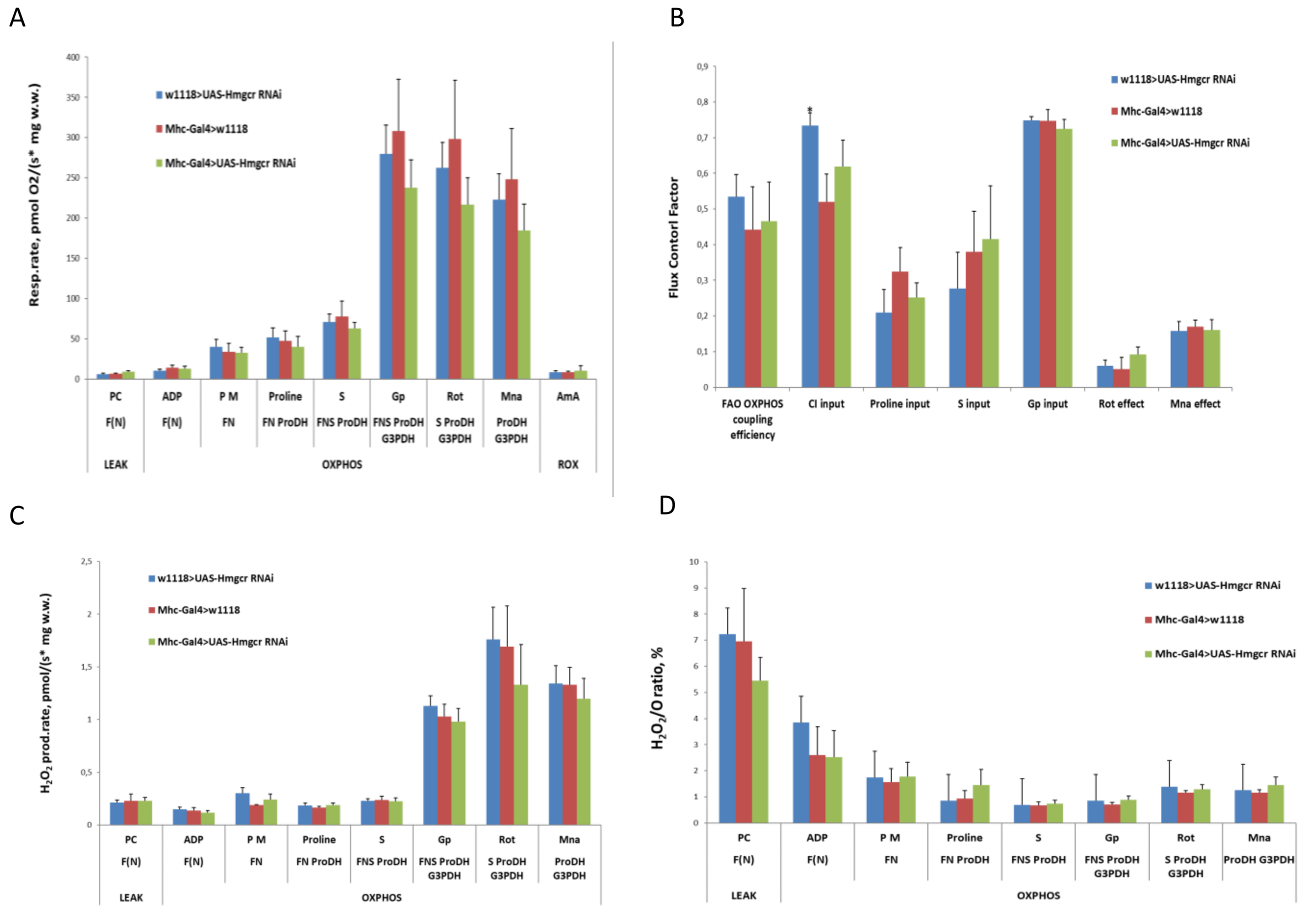

Supplementary Fig. 5: Hmgcr knockdown in skeletal muscles does not result in FAO impairment. The thorax muscles were dissected for the respirometry assay, where *Mhc-Gal4>UAS-Hmgcr RANi* is the *Hmgcr* knockdown flies, while *w1118>UAS-Hmgcr RNAi* & *Mhc-Gal4>w1118* are the control groups. Each bar represents the mean ± SEM, and the statistical significance was calculated using One-Way ANOVA with Bonferroni's multiple comparisons test whereby *, P≤0.05 and N=5. PC, palmitoylcarnitine; ADP, adenosine diphosphate; PM, pyruvate and malate; P, pyruvate; S, succinate; Gp, Glycerol-3-phosphate; U, uncoupler; Rot, rotenone; AmA, antimycin A; F, fatty acid oxidation-dependent pathway; N, NADH pathway; LEAK, substrate-dependent state; OXPHOS, oxidative phosphorylation-dependent state; ROX, residual oxygen consumption; H2O2, hydrogen peroxide; G3PDH, glyceraldehyde-3-phosphate dehydrogenase.
